# Design and Development of a Wearable Assistive Device Integrating a Fuzzy Decision Support System for Blind and Visually Impaired People

**DOI:** 10.3390/mi12091082

**Published:** 2021-09-07

**Authors:** Yassine Bouteraa

**Affiliations:** 1 Department of Computer Engineering, College of Computer Engineering and Sciences, Prince Sattam bin Abdulaziz University, Al-Kharj 11942, Saudi Arabia; yassine.bouteraa@isbs.usf.tn; 2 Control and Energy Management Laboratory (CEM Lab.), Ecole Nationale d Ingenieurs de Sfax (ENIS), Institut Superieur de Biotechnologie de Sfax (ISBS), University of Sfax, Sfax 3038, Tunisia

**Keywords:** navigation aid, blind and visually impaired people, sensor data fusion, fuzzy classifier, assistive technology, wearable devices

## Abstract

In this article, a new design of a wearable navigation support system for blind and visually impaired people (BVIP) is proposed. The proposed navigation system relies primarily on sensors, real-time processing boards, a fuzzy logic-based decision support system, and a user interface. It uses sensor data as inputs and provides the desired safety orientation to the BVIP. The user is informed about the decision based on a mixed voice–haptic interface. The navigation aid system contains two wearable obstacle detection systems managed by an embedded controller. The control system adopts the Robot Operating System (ROS) architecture supported by the Beagle Bone Black master board that meets the real-time constraints. The data acquisition and obstacle avoidance are carried out by several nodes managed by the ROS to finally deliver a mixed haptic–voice message for guidance of the BVIP. A fuzzy logic-based decision support system was implemented to help BVIP to choose a safe direction. The system has been applied to blindfolded persons and visually impaired persons. Both types of users found the system promising and pointed out its potential to become a good navigation aid in the future.

## 1. Introduction

According to the WHO [1], 1.3 billion people suffer from visual impairment, of which the numbers of people with mild visual impairment, moderate to severe visual impairment, and the blind are 188.5 million, 217 million, and 36 million, respectively. Those affected have their autonomy threatened in many daily tasks, with the emphasis being placed on those that involve moving through an unknown environment. A state of decreased visual perception is called blindness. It is composed of two main classes: partial blindness, which means a lack of integration in the growth of the optic nerve, and total blindness, which is the full absence of visual light perception. Accordingly, BVIP use certain techniques and tools to regain their autonomy. To this end, and from a functional point of view, the design of this type of assistance requires that the information be provided in a non-visual form and in a simple and rapid manner. From a design point of view, the device itself should be light, easy to use, and not a source of nuisance or disruption.

Several recent works review the development of navigation systems for BVIP, presenting the past works, challenges, and open problems [2,3,4,5]. Various areas of R&D fields have received a great deal of attention to meet the needs of BVIP, such as mixed reality [6,7,8], smartphones [9,10], and wearable devices [11,12,13,14]. Efforts have been dedicated for more than two decades to the search for technological solutions to guide the visually impaired. Indeed, a system to warn blind or visually impaired travelers that they have entered a potentially dangerous area near public transport hubs was developed within Johns Hopkins University [15]. A hand-held device that allows visually impaired people to move around was patented in 2016. The developed device recognizes the locations of objects and determines the path through them. A built-in speaker provides audio instructions to a user. However, this system does not consider drilling or bumps [16]. A low-cost system based on ultrasonic sensors and an audible warning system was designed to guide the visually impaired in [17]. A smart white cane based on cloud computing and Internet of Things (IoT) wireless scanners was designed to facilitate indoor navigation [18]. Most of today’s solutions use new technologies such as artificial intelligence and connected objects. A model based on fuzzy logic and IoT systems for the safe mobility of blind people was proposed as a solution for indoor navigation [19]. A wearable navigation device combining semantic visual SLAM (Simultaneous Localization and Mapping) and a powerful mobile computing platform was proposed to guide navigation for blind people [20]. Various recent navigation tools to help visually impaired people are presented in [21]. Generally, the cane is the most popular designed system dedicated to navigation for blind people, including the white cane [22], the smart cane [23], and the laser cane [24]. However, the cane-based navigation system has several constraints concerning its size and weight. In addition, this solution presents limitations to recognizing obstacles, and it is also difficult to use the cane in public places. Inspired by these constraints, many techniques have been developed based on smart sensor technology and digital data processing to enhance the mobility of BVIP and help them to move freely in an environment regardless of its dynamic changes. Based on the related literature, these techniques are classified into two main types: sonar input [25,26] and camera input systems [27,28,29,30]. Generally, the sonar systems are based on ultrasonic, laser, and infrared signals as inputs, which can provide a range of information of an obstacle. However, this method cannot provide precise information, such as shapes and dynamic obstacles, and also cannot make judgments regarding the travel orientation. On the other hand, camera-based navigation systems provide useful information about the state of obstacles by capturing all environmental information and assist the user to select the preferred travel path. However, constraints are imposed by the larger size and relatively higher cost, and these systems require more complex algorithms for image processing, which may impose the use of powerful computing machines. Many systems integrate cameras, infrared, and depth sensors based on the Kinect smart sensor in order to identify hurdles and suggest a safe path [31,32,33]. However, the improvement of processing power requires a durable and high-power supply, which can cause excessive weight. Various concepts transforming spatial data into sound were developed by using an acoustic information presentation approach [34,35], speculating on their applicability as sensory substitution devices (SSDs). However, the main drawback of such systems is that acoustic feedback causes distraction and interferes with the user’s normal hearing activities. On the other hand, tactile feedback has been used as a navigational assistive interface for the visually impaired to avoid user distraction with musical tones [36,37]. For these systems, such drawbacks are found. Indeed, the main drawback in the touch stimulation systems is that the information can be complex and slow to understand. Additionally, most interfaces using this tactile stimulation are aimed at fingertips and palms. They are often used in conjunction with basic assistance devices such as a white cane and consequently quickly cause body fatigue and user rejection since they require constant hand interaction.

**Figure 1:**
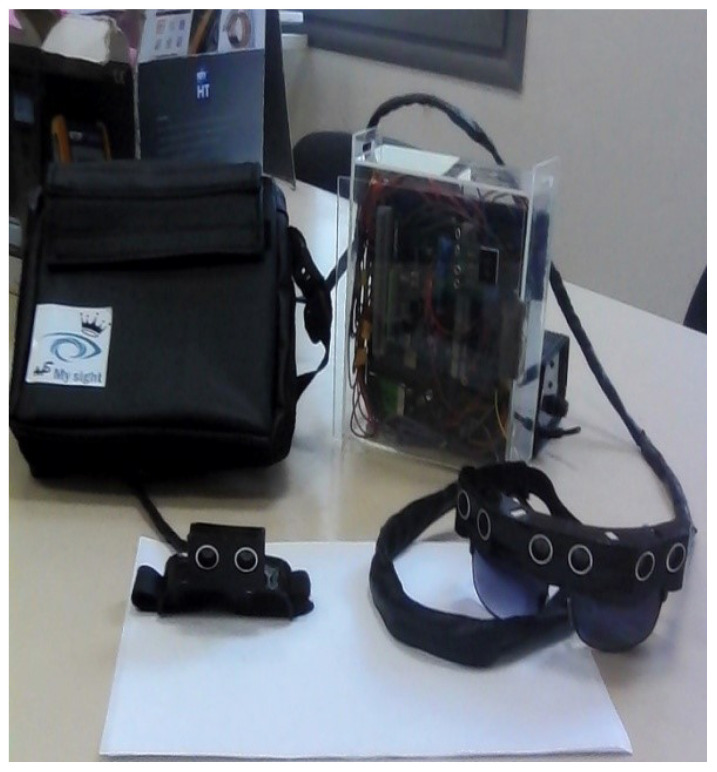
The different components of the designed system.

In order to overcome these drawbacks, the developed system uses a speech generation device to inform the user about the algorithm’s decision via a simple integrated earphone. Indeed, only important information is sent to the user, including the path’s safety state, the power level of the system (if any), and the desired safe path. Thus, the user may not receive feedback from our system if all is well. In addition, real-time depth information is converted into vibrations to minimize distraction and therefore not interfere with the user’s normal hearing activities. In conclusion, the use of speech is restricted to the scenarios when the navigation path is complicated and requires a computer-based decision-making approach. Indeed, we propose a new design of a wearable device which converts obstacle depth into speech and vibration in real-time. The distance from the sensor is translated into vibration intensity and the subject is informed of the final decision by the embedded fuzzy controller using speech technology. As illustrated by Figure 1, the proposed system is composed of three main components: an ROS-based embedded controller, an eyeglass frame containing three ultrasonic sensors with the related vibrator modules for obstacle detection, and a similar accessory that can be worn in the hand for ramp and up–down barrier detection. The vibrator modules are used as tactile interfaces that stimulate the plantar surface with vibrations. Therefore, this work offers a guidance system for BVIP to assist them in walking in indoor and outdoor surroundings.

This article is organized as follows: materials and methods are presented in Section 2, providing the mechanical design, including the prototyping technique and the electronic design of the embedded controller with the implemented obstacle detection approach. The experimental results of the preliminary tests with healthy and blind participants are discussed in Section 3. Finally, we conclude with a summary of the work and a perspective.

## 2. Materials and Methods

### 2.1. Design Requirements

A major flaw in the design of navigation systems for BVIP appears to lie in a set of reiterated deficiencies in the knowledge of users’ needs, capabilities, and limitations. The main three functionalities of navigation systems for the blind are as follows:

- The first point concerns positioning systems intended to provide location information. The choice of these components during the design depends mainly on the requirements of the desired solution and the application environment (interior/exterior), and in light of these two factors, the choice of the appropriate technology is made taking cost into account;
- The second point is environmental monitoring, which is very important in these types of applications to detect static and dynamic obstacles in particular;
- The third is the user interface, which relies on data collected from the environment and navigation and communicates this information to blind users to guide them while navigating.

#### 2.1.1. Unit Description

**Figure 2:**
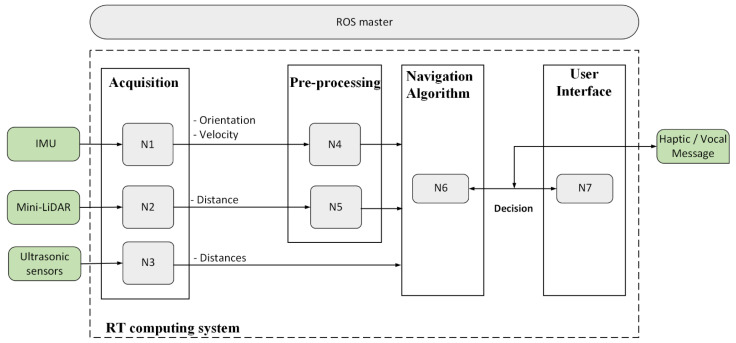
High level architecture for the navigation system.

The real-time navigation system is mainly based on reliable sensors, real-time processing boards, and a user interface. It uses sensor data as inputs and outputs the desired safe orientation of the user. The developed unit includes mini-LiDAR, IMU, and ultrasonic sensors as inputs. In addition, a mixed haptic–vocal interface is provided as a user interface. The power supply is designed to accommodate a 12 V DC input. Figure 2 illustrates an overview of the developed real-time navigation unit. The system is composed of three subsystems: the eyeglass, containing three ultrasonic sensors with the related vibrator modules assembled in a headband; the wearable hand accessory, containing the mini-LiDAR; and the related vibration module and the control and power systems, embedded inside a handbag that is portable for the user. Regarding the preprocessing stage, both the IMU and the mini-LiDAR were considered. The following actions were applied in the processing stage:

- Compute the velocity from the acquired position data (from the UM7);
- Extract the Yaw value from the magnetometer data, applying a smoothing filter for speed and orientation data (from the UM7) and the distance (from the mini-LiDAR);
- Normalization of the rectified data dedicated for fuzzy classifier inputs by mapping all data to [0, 10].

The low-level architecture is based on parallel processing. Task scheduling in parallel processing is a technique that divides the process into multiple processes and executes them concurrently with the use of more than one CPU or processor. This architecture provides concurrency, reduces timing constraints, and allows researchers to solve larger problems. It also opens up the possibility of the integration of additional peripherals. The hardware architecture (Figure 2) consists mainly of sensors, real-time boards, and a data transferring user interface.

#### 2.1.2. Navigation System Architecture

The navigation system architecture is based on the ROS, which is an operating system that is used worldwide and provides libraries, tools, and an easy environment to develop robotic applications. In addition, the adoption of the ROS in the industry is continuously growing. The ROS is an open-source software platform dedicated to robotics development. It stands out as a result of its very rich libraries, such as 2D laser SLAM, object recognition based on a 3D point cloud, Adaptive Monte Carlo, Extended Kalman Filter localization, etc., as well as tools for visualization (rviz), the recording of experiments, and reading data offline (rosbag), etc. It has a modern architecture that allows it to connect with different devices and applications and to run on different machines in parallel and in real-time. The proposed architecture for the navigation system is given in Figure 2. In this architecture, several nodes were developed to perform sensor data acquisition, pre-processing, and navigation. The ROS master connects between the different nodes and delivers the decision as a mixed haptic–vocal message.

The low-level architecture is based on parallel processing. Task scheduling in parallel processing is a technique that divides the process into multiple processes and executes them concurrently with the use of more than one CPU or processor. This architecture provides concurrency, reduces timing constraints, and allows the system to solve larger problems. It also opens up the possibility of the integration of additional peripherals. To satisfy real-time requirements, the system tasks (acquisition, processing, and decision transfer) must be accomplished within the sampling time T depending on the selected sensors, processors, and interfaces. The hardware architecture (Figure 2) consists mainly of dedicated sensors, real-time boards, and a data transferring user interface.

#### 2.1.3. Mechanical Design

As discussed previously, the physical constraints of the wearable guidance device address the need for three interrelated factors: ergonomics, performance, and comfort. In this section, according to these factors, the mechanical architecture design, as well as the electronic part, is presented. Indeed, our device is presented as a simple glasses frame provided with a special mechanical structure supporting three ultrasonic sensors in three views, combined with a hand band for path scanning based on a LiDAR sensor. The mechanical structure was designed with well-studied characteristics. For more comfortable guidance, the assistive device should be a light and easy to use. In addition, the device must have a small size and weight for more comfortable guidance. As presented in [32], several rapid prototyping (RP) techniques are useful in the medical field. In particular, the use of 3D printer technology is expanding in the guidance devices field. Therefore, the use of an additive manufacturing process allows a high level of customization to be attained, thus requiring only the geometric model of the wearable guidance system to be realized (3D printed). The three main steps of the mechanical manufacturing of the designed device using 3D printing technologies can be outlined as follows:

- Modeling of the 3D geometry of the desired wearable device using CAD software;
- Processing of the acquired data through dedicated software;
- Realization of the designed device using a 3D printer.

**Figure 3:**
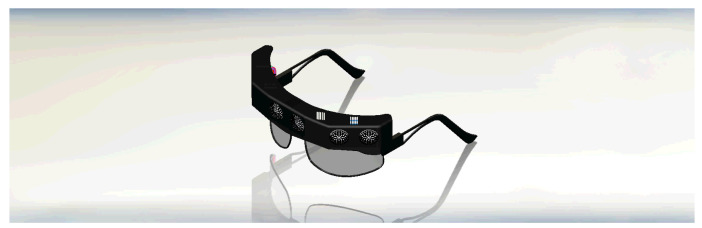
The designed frame with its integrated sensors.

**Figure 4:**
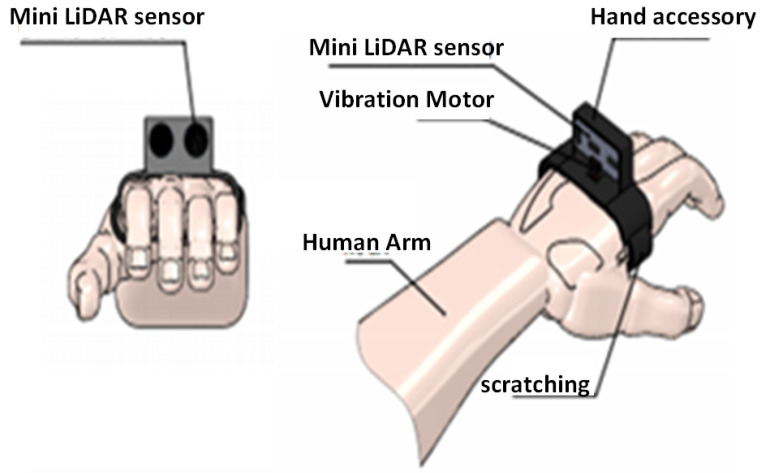
Wearable hand accessory to detect lower objects and the corresponding 3D geometric model.

The model design of the guidance device was created using SOLIDWORKS CAD software and subsequently printed using a 3D printer. Indeed, the possibility of realizing a highly customized wearable device received a boost thanks to the widespread diffusion of low-cost 3D printing technologies. Figure 3 illustrates the frame-based wearable device and the integrated sensors dedicated to upper obstacle detection. On the other hand, Figure 4 describes the hand band accessory dedicated to the detection of lower obstacles, shapes, and cavities.

#### 2.1.4. Electrical Design

Here, we present the electronic part responsible for powering and controlling the different components of the designed system. The control box, placed in the wearable bag, is responsible for obstacle detection and decision making. The main components of the control box are composed of three major blocks for acquisition, processing, and alerting. Firstly, the acquisition block is based on three ultrasonic sensors placed in the glasses frame support for upper obstacle detection. Indeed, an HC-SR04 ultrasonic sensor is used. This sensor uses sonar to determine the obstacle depth. Thus, it offers excellent non-contact range detection with high accuracy and stable readings. From 2 cm to 400 cm, this sensor offers a wide range of scanning areas. It is fully integrated, containing an ultrasonic transmitter and receiver module. These sensors are directly plugged into the Arduino slave board. On the other hand, a mini-LiDAR sensor is implemented in the hand band accessory, covering the lower location. A LiDAR module is implemented to measure distances from 0.3 to 12 m. This module communicates directly with the Beagle Bone Black via a 3.3 Vdc UART link as a slave node. Distance measurement is based on the Time-of-Flight method, which allows distances to be measured precisely using infrared pulses. The key reasons for using the LiDAR sensor on the hand band are as follows:

- The importance of the lower area in the navigation of visually impaired and blind peoples. Indeed, the ground can include either a low obstacle or a hole, which are the main factors affecting the safety of the navigation path. This requirement means that the used sensor must cover a wide scanning area with high precision and accuracy;
- The shape of the ground. Indeed, identifying the slope of the ground is indispensable for safe navigation. This requires a sensor that can detect the distance of the barrier even if the shape is complicated;
- The ground scanning method must be as continuous as possible during navigation. This means that the hand is the best location for the sensor to be embedded. This constraint requires that the related sensor must be fully integrated with a small size and weight.

Although the previous sensors are used mainly for environment identification, knowledge of the speed of the navigation profile of the visually impaired pedestrian is also very important. The navigation profile includes position, speed, and acceleration. The useful information for safety assessment is the speed of the pedestrian. To meet this requirement, the UM7 was selected. Indeed, it is a third-generation Attitude and Heading Reference System (AHRS) that improves on the performance of the MEMS technology. The UM7 integrates a triaxial accelerometer, rate gyro, and magnetometer. Estimated attitude and heading data are improved based on an integrated Extended Kalman Filter (EKF). The UM7 is considered a slave node that communicates with the master board via UART communication. The main features of the selected IMU are as follows:

- Higher gyro bias stability and lower noise;
- A new communication architecture for improved flexibility;
- UTC time-synchronization with external GPS;
- Support for third-order temperature compensation on all sensors.

To alert a pedestrian when an obstacle is positioned inside the defined scanning area, two types of alerts are used: a mini motor as haptic feedback using vibration alert, and a voice message to send the final decision to the pedestrians via an external headset or earpiece. Firstly, due to their small size and enclosed vibration mechanism, micro vibrating motors are a popular choice for many different integrated solutions. They are great for haptics, particularly in handheld instruments where space can be at a premium, such as in mobile phones, portable instruments, and medical applications. In our case, the vibration motor is used not only for vibration alerting but also for haptic feedback. Indeed, vibration alerting tends to be a simple on/off alert informing the visually impaired person that an obstacle exists. Moreover, haptic feedback uses advanced vibration patterns and effects to convey complicated information to users. Progressing from vibration alerting to haptic feedback, the depth of a detected obstacle, computed by the related ultrasonic sensor, is mapped to vibration intensity. Therefore, the visually impaired person can estimate the distance of the obstacle and determine the dynamics of the unknown obstacle. Since the vibration motors are designed to be easy to mount, a headband and hand band are designed including the vibration motors.

Moreover, the speech synthesis shield is used for easy communication with the ROS master as a slave node via UART communication using the ROS-serial feature. It uses an XFS5051CE speech synthesis chip with a high degree of integration. It supports both Chinese and English languages. Three popular interfaces are available for use with this shield: UART, I2C, and SPI. The speech synthesis shield is plugged into the master board, allowing the navigation support system to send the decision with a voice message. The whole navigation system is powered by a 12 V 18 Ah Li-ion rechargeable battery providing a long autonomy (about 7 working hours in the worst-case scenario) and meets physical constraints. Noting that the test of the power level of the vibrating motor is done directly on the Arduino analog input, this test allows the measurement of the voltage and for any drop in voltage to be accurately detected. Indeed, a voltage drop at a threshold level (3.5 V) brings the system into an economical mode, which is characterized by a decrease in the maximum duty cycle of the vibration intensity. In addition, the subject will be informed by the speech module that the vibration motor power is at a low level by sending the voice message “low vibration battery” each minute. If the user does not replace the battery and the high generated vibration intensity becomes very weak, the vibrating option is automatically disabled and only the voice message is considered for communication. Because the Arduino’s microcontroller cannot receive a voltage greater than 5 V as an analog input, a divider bridge is designed to allow the Arduino’s power to be tested. Indeed, the power level of the battery decreases according to its discharge; therefore, the battery should be connected with a voltage divider and the resulting voltage mounted on the analog input of the Arduino board to calculate the percentage of charge remaining according to the measured voltage. If the supply voltage drops to a critical value (6 V), the guidance system will be switched to another economic mode. In this mode, the vibration motors will be disabled. In addition, a prediction algorithm will be launched to estimate the remaining time in which the supply voltage remains greater than 5 V. The user will be informed of the remaining operating time each minute.

### 2.2. Navigation Approach

**Figure 5:**
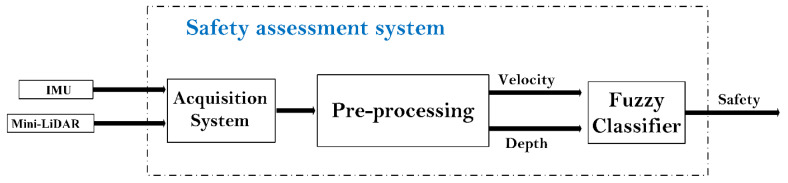
Safety assessment system using sensor data fusion and fuzzy classifier.

The control system architecture is based on a Beagle Bone Black (BBB) board and an Arduino Due. Indeed, the BBB master board is dedicated to data fusion and decision making, while the Arduino board is integrated as a slave board responsible for ultrasonic sensor acquisition and an interface for vibration motor actuation. Indeed, this integrated subsystem can be considered as an obstacle detection module based on the ultrasonic sensors and the haptic feedback. Although the three ultrasonic sensors dedicated for high obstacle detection are acquired and processed directly by the Arduino board, which actuates the related vibration modules by mapping the measured distance to vibration intensity, the measured values are sent to the master board for data fusion and decision making. Communication between master and slave is ensured via the UART protocol. Beginning by acquiring the geometric data from the different implemented ultrasonic sensors, and based on these measures, the existence of high-level obstacles in the defined scanning area is determined. If any obstacle is scanned in the forward path, it is noted as being straight ahead, and the new measures will be acquired from sensors. On the other hand, if an obstacle is detected by any implemented sensor, the system will alert the user to change their direction to a safer path. To determine the safe path for the user, a fuzzy logic classifier is designed based on the hand band sensor and an IMU. Indeed, the hand band mini-LiDAR sensor is dedicated to detecting the shape of the ground including the depth data. The IMU is implemented to estimate the velocity of the human. Human velocity and depth data are processed and fused as inputs of the fuzzy controller, which outputs the path risk assessment. The user is informed of the decision of the controller by a vocal message. The proposed fuzzy system uses the human velocity and the ground depth to estimate the safety level during navigation, as shown in Figure 5.

This makes the developed system a smart decision-making system compared to on–off switching systems.

The rules database is implemented using LabVIEW software. Indeed, 15 if/then rules are implemented based on the database presented in Table 1.

A complete list of the implemented evaluation rules can be enumerated as follows:

- 1. IF “depth” IS “very low” AND “human velocity” IS “low” THEN “safety level” IS “very low”;
- 2. IF “depth” IS “low” AND “human velocity” IS “low” THEN “safety level” IS “low”;
- 3. IF “depth” IS “medium” AND “human velocity” IS “low” THEN “safety level” IS “medium”;
- 4. IF “depth” IS “high” AND “human velocity” IS “low” THEN “safety level” IS “medium”;
- 5. IF “depth” IS “very high” AND “human velocity” IS “low” THEN “safety level” IS “high”;
- 6. IF “depth” IS “very low” AND “human velocity” IS “medium” THEN “safety level” IS “low”;
- 7. IF “depth” IS “low” AND “human velocity” IS “medium” THEN “safety level” IS “medium”;
- 8. IF “depth” IS “medium” AND “human velocity” IS “medium” THEN “safety level” IS “medium”;
- 9. IF “depth” IS “high” AND “human velocity” IS “medium” THEN “safety level” IS “high”;
- 10. IF “depth” IS “very high” AND “human velocity” IS “medium” THEN “safety level” IS “very high”;
- 11. IF “depth” IS “very low” AND “human velocity” IS “high” THEN “safety level” IS “medium”;
- 12. IF “depth” IS “low” AND “human velocity” IS “high” THEN “safety level” IS “medium”;
- 13. IF “depth” IS “medium” AND “human velocity” IS “high” THEN “safety level” IS “High”;
- 14. IF “depth” IS “high” AND “human velocity” IS “high” THEN “safety level” IS “very high”;
- 15. IF “depth” IS “very high” AND “human velocity” IS “high” THEN “safety level” IS “very high”.

## 3. Case Study

**Figure 6:**
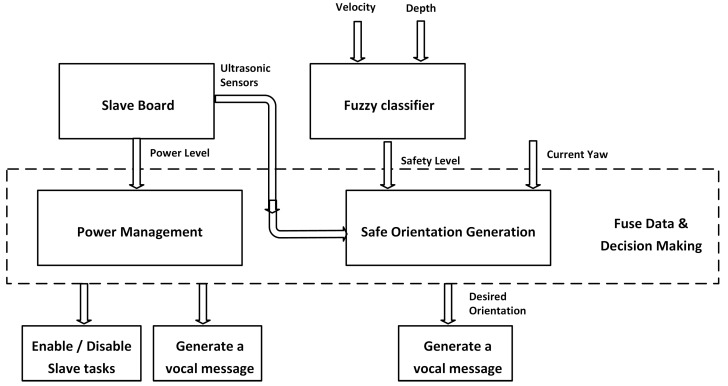
Block diagram of the main controller.

**Figure 7:**
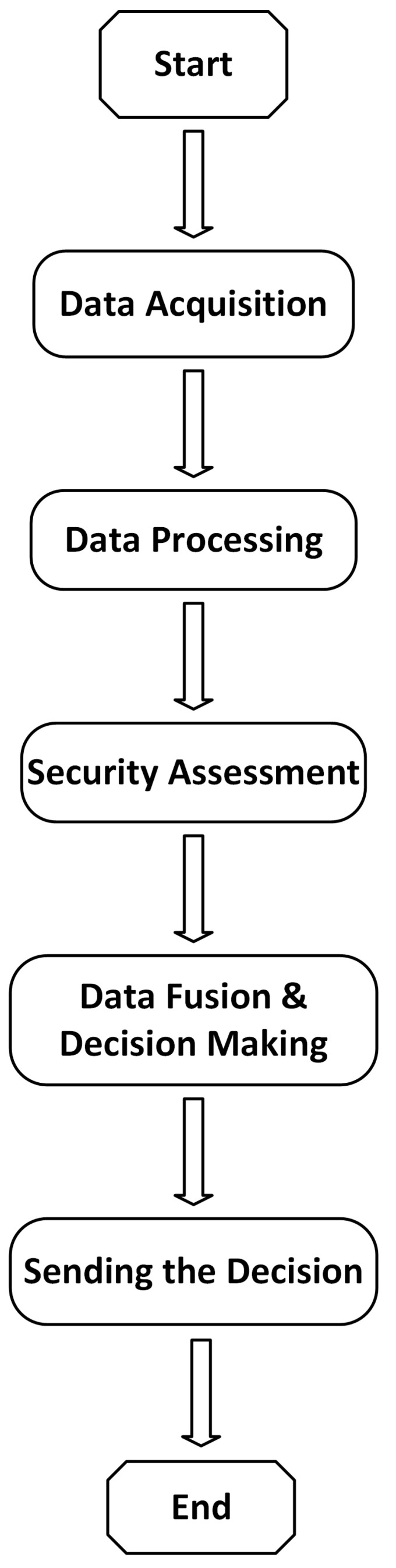
Operating flowchart.

The main algorithm of the master board is shown in Figure 6, illustrating the main inputs/outputs of the proposed approach. The operating flowchart of the navigation system is shown in Figure 7. According to this flowchart, the main controller fuses data and decides the safe direction. Although the three ultrasonic sensors, dedicated for high obstacle detection, are acquired and processed directly by the Arduino board, which actuates the related vibration modules by mapping the measured distance to vibration intensity, the measured values are sent to the master board for data fusion and decision making. In addition, the state of the power level is also sent to the master board, which determines the suitable functional mode and estimates the remaining working time. The master board generates the suitable vocal message and sends it to the user. On the other hand, the master board is responsible for power management and can enable or disable the slave board according to the power state.

The designed guidance system was tested using one visually impaired person with some experience with intelligent guidance systems, such as the smart cane. The tests were carried out in indoor and outdoor environments and with different procedures and scenarios to verify all aspects, ranging from pure functionality to the higher level of performance and reliability. In this part, we present one trial test that covered most of the aspects of the developed navigation-aided system. The navigational performance of the participant was observed as a function of the number of collisions during the walk. On the other hand, safety performance was estimated based on the ability of the system to detect dangerous scenarios and the correctness of the decisions about the desired safe path. Tests were performed in a simulated indoor environment imitating both indoor and outdoor features. The desired path was planned to be as long as possible in order to test the power performance.

The first step consisted of installing the navigation system peripherals, including the vibration modules and the earphone. Regarding the wearing of the designed system, the user could easily wear the navigation system by fixing each module based on a scratch attachment. The system can be divided into three subsystems, which can be listed as follows:

- The eyeglass, containing three ultrasonic sensors with the related vibrator modules, assembled in a headband and attached via scratch;
- The wearable hand accessory, including the mini-LiDAR and the related vibration module, which is also attached by a simple scratch;
- Control and power systems embedded inside a handbag that is portable by the user.

The next step comprised the verification of the correspondence of the haptic feedback with the obstacle depth. This was done by using a mobile obstacle and verifying the vibration intensity. Another concept to be tested was the correctness of the generated vocal message according to the sensed vibration intensity. Finally, the user was asked to increase their speed within the dynamic obstacles in order to prove the safety of the algorithm. Table 2 presents the preliminary results as a proof-of-concept.

## 4. Experimental Results

The navigation support system was evaluated in a preliminary user study with some healthy participants and then with BVIP subjects.

### 4.1. Experimental Setup and Tests with Healthy Participants

**Figure 8:**
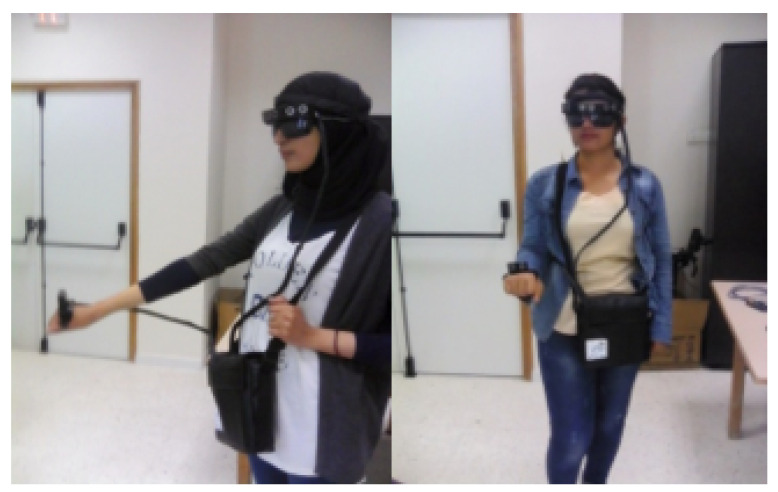
Preliminary tests and validation with healthy participants.

For experimental evaluation, an experiment with some healthy participants was conducted to examine the ability of the navigation support system to guide healthy subjects and to detect both static and dynamic obstacles. For this experiment, two subjects were invited to test the developed system as described in Figure 8. Before beginning, a preliminary adjustment was conducted. Both vibrating bands needed to be securely attached to the head and hand, ensuring the ability of the signaling system to inform the subject of the existence of an obstacle in the defined scanning area using the haptic feedback. Firstly, the healthy participants were asked to simply move in an indoor environment that contained several types of obstacles. In the rest state, the system reacted only to dynamic obstacles. This test was performed to ensure that the response time was sufficient to detect the presence of a dynamic obstacle and also to prove that the implemented controller can make the correct decision according to the dynamic behavior of an obstacle. Secondly, the participants performed some special movements to validate the obstacle detection approach in different types of navigation. After that, both tests were repeated for reassessment in the outdoor environment.

The experiment with healthy participants had two objectives: first, to prove that the developed navigation system could accommodate a subject with high security for both outdoor and indoor environments; and second, to determine whether the developed control system could, at least, understand the behavior of the dynamic obstacles and can make the optimal decision about safe orientation. Based on these tests, the next phase was to certify the developed navigation support system in a real environment by applying it to blind and visually impaired subjects.

### 4.2. Experimental Setup and Tests with Visually Impaired Subjects

In the experimental setup, we set three paths in indoor and outdoor environments, respectively, to evaluate the navigation system including the safety assessment system. Indeed, we chose an office area as the indoor testing environment and simulated the scenarios that a BVIP would usually encounter in outdoor scenarios. In the indoor environment, we placed a fixed number of obstacles on the four paths, whereas in the outdoor environment, we added some dynamic obstacles that the participants encountered when they were performing the test, resulting in mixed static–dynamic obstacles in the desired path. Note that a dynamic obstacle was simulated as an object moving at low speed in a random path regardless of the size and shape of the obstacles. On the other hand, to test the safety assessment system, some customized obstacles were integrated into the desired path, imitating complex shapes of the ground, which could thus be estimated as an unsafe path. A preliminary assessment of the designed guidance system was performed on 15 visually impaired participants. Before the test, all participants were given a brief explanation of the designed navigation aid system until they understood the overall procedure of the experiment and how to use our system. Given its great flexibility and low weight characteristics, all subjects provided good feedback on their first impressions of the device. They were then asked to navigate, following the desired paths with the help of either our designed system or a conventional white cane. Using a cane, the user could feel obstacles in their path without stepping on them, but not all obstacles were accessible with the cane. Indeed, the descending ramps and the cavities of the ground were not perceptible with the cane; the same was true for suspended objects such as wires. In addition, users could not scan all walking spaces at ordinary walking speed, and even obstacles in the middle of their path, such as a chair or table, could produce a collision with increasing speed. To compare our system with the white cane fairly, subjects were asked to follow the desired path including the same static and dynamic obstacles. Then, we noted the average walking time and the number of collisions to evaluate the performance of our system, especially the ability of our system to detect risks and find a safe orientation.

According to the results presented in Table 3, we can see that the subjects that used our designed navigation system exhibited less walking time than those using the conventional white cane for both outdoor and indoor environments, demonstrating the ability of our system to navigate users efficiently when they are in unknown environments. Moreover, the subjects had more collisions when using the white cane, as the obstacles hanging low and in mid-air were hard to detect for the white cane, especially when the obstacle shape was complicated; whereas with our system, the subjects could avoid such collisions even with dynamic obstacles. Table 4 illustrates the number of collisions with dynamic obstacles versus static obstacles and for high obstacles versus medium obstacles versus low obstacles for both methods. However, in the paths of the outdoor environment, the subjects with our system still experienced a few collisions due to the dynamic behavior of the obstacles and because the small-size objects were difficult to identify, as they had a depth value approximating the depth of the ground. Although the participants collided with some lower obstacles, the small objects did not harm the participants. Indeed, these collisions were mainly due to the user’s performance and the degree of impairment, which can be adopted in the most human-centered approaches, in which the user collaborates with the computer system to analyze and apply decisions. Concerning the dangerous obstacles in the paths—generally, a gap and ramp on the ground—our system showed a good ability to detect these obstacles and avoid a dangerous path. Therefore, the proposed system was verified to be secure for navigation. After the real-world test, in order to give useful feedback about our designed system, seven subjects were asked some simple questions about the following factors:

- The easiness of wearing and the portability of the device;
- The provided assistance to move in unknown paths and unfamiliar environments;
- How safe they felt when using our device;
- The real-time decisions about the safety of the path and the avoidance of the dynamic objects;
- Suggestions for product improvement.

The questionnaire is shown in Table 5, and all users answered that the system was useful and helped them to perceive semantic surrounding information. Indeed, constructive feedback was presented from the most qualified subjects who had a good knowledge of the trends in the guidance systems dedicated for visually impaired people. The suggested improvements are well appreciated from a technological point of view. However, from a product development point of view, the recommended functionalities, such as face/object recognition/identification, would be very costly. The additional cost of the hardware executing the face/object recognition may not be supported by the buyer. Our approach is to design a low-cost navigation-aided system that can be used by a wide range of people and providing a good value for money.

## 5. Conclusions

A navigation system for the blind and visually impaired was developed and tested in indoor and outdoor environments. The proposed navigation system consists of an eyeglass frame, a hand band accessory, and an ROS-based embedded controller. The ROS was chosen as the operating system in order to guarantee real-time functionality as well as a parallel architecture that is open to other extensions. The Beagle Bone was selected as the main CPU supporting the ROS architecture. In addition, it met the computational requirements by performing the required tasks within the defined sampling time. The developed architecture was equipped with advanced exteroceptive and proprioceptive sensors providing data on navigation parameters (position, speed, acceleration, and direction) and obstacles, whether on the ground or in front of the user. A safety assessment system was implemented using sensor data fusion and a fuzzy classifier to determine safe paths for users. The integration of the ROS into the developed system made it possible to deal with the various tasks of acquisition, obstacle avoidance, and real-time guidance. Compared to the cane, the developed system showed a better performance mainly in the detection of dangerous obstacles. Some components and functionality need to be incorporated into future work such as GPS and object identification. The proposed guidance system was tested in real-time by BVIP in different indoor and outdoor environments and has shown promising results. Actual tests with BVIP showed that the designed navigation aid system had a positive impact on obstacle detection, recognition of ramps and cavities, and interaction with dynamic obstacles. The device is scalable, and future work aims to integrate global location tracking and shortest route routing, which are very important features to help visually impaired people reach a target destination and understand the natural world around them.

## Figures and Tables

**Table 1:** Rules database.

		Velocity		
		Low	Medium	High
Depth	Very Low	Very Low	Low	Medium
	Low	Low	Medium	Medium
	Medium	Medium	Medium	High
	High	Medium	High	Very High
	Very High	High	Very High	Very High

**Table 2:** Proof-of-concept results.

Simulated Environments	Haptic Feedback	Vocal Message	Safety Estimation	Power Management	Real-Time Decision Making	Safe Path Generation
Indoor	Reliable	Reliable	Good	Excellent	Yes	Good
Outdoor	Reliable	Acceptable	Acceptable	Excellent	Yes	Acceptable

**Table 3:** Results of navigation assisted by a cane and with the designed system.

Scenario	Path	Length (m)	Obstacles		Dangerous Obstacles	Average Time (s)		Total Collisions		Dangerous Obstacles Detection		Trust Safe Path Generation	
			Static	Dynamic		Ours	Cane	Ours	Cane	Ours	Cane	Ours	Cane
Indoor	Path1	32	3	0	0	114	121	0	0	-	-	-	-
	Path2	44	5	1	0	165	178	0	4	-	-	-	-
	Path3	54	11	2	1	215	244	0	8	1	1	Yes	No
Outdoor	Path4	241	28	10	2	569	681	3	28	2	2	Yes	No
	Path5	316	39	16	5	754	988	5	40	4	3	Yes	No
	Path6	411	55	22	11	911	1058	12	61	9	5	Yes	No

**Table 4:** Collision with different types of obstacles and comparison with the white cane solution.

Total Collisions		Obstacles Types				Collisions with High Obstacles		Collisions with Middle Obstacles		Collisions with Ground Obstacles and Cavities	
Ours	Cane	Static		Dynamic		Ours	Cane	Ours	Cane	Ours	Cane
		Ours	Cane	Ours	Cane						
0	0	0	0	0	0	0	0	0	0	0	0
0	4	0	3	0	1	0	3	0	0	0	1
0	8	0	6	0	2	0	6	0	1	0	1
3	28	0	20	3	8	1	14	0	4	2	10
5	40	1	25	4	15	2	22	0	5	3	13
12	61	2	42	10	19	4	32	1	8	7	21

**Table 5:** The question sheet.

Participants	Totally Blind or Partially Sighted	Easiness	Assistance	Safety	Real-Time	Suggestions
Participant 1	Partially sighted	Yes	Yes	Yes	Yes	The device should be more compact
Participant 2	Partially sighted	Yes	Yes	Yes	Yes	–
Participant 3	Totally blind	Questionable	Yes	Questionable	Yes	Add face recognition
Participant 4	Totally blind	Yes	Yes	Questionable	Yes	Add object identification
Participant 5	Totally blind	Yes	Yes	Yes	Yes	Add character recognition, cash recognition
Participant 6	Partially sighted	Yes	Yes	Yes	Yes	Reduce cost of product
Participant 7	Partially sighted	Questionable	Yes	Yes	Yes	Add a GPS

## References

[1] Bourne R.R., Flaxman S.R., Braithwaite T., Cicinelli M.V., Das A., Jonas J.B., Keeffe J., Kempen J.H., Leasher J., Limburg H. (2017). Magnitude, temporal trends, and projections of the global prevalence of blindness and distance and near vision impairment: A systematic review and meta-analysis. Lancet Glob. Health.

[2] Real S., Araujo A. (2019). Navigation systems for the blind and visually impaired: Past work, challenges, and open problems. Sensors.

[3] Manjari K., Verma M., Singal G. (2020). A survey on assistive technology for visually impaired. Internet Things.

[4] Tapu R., Mocanu B., Zaharia T. (2018). Wearable assistive devices for visually impaired: A state of the art survey. Pattern Recognit. Lett..

[5] Khan S., Nazir S., Khan H.U. (2021). Analysis of Navigation Assistants for Blind and Visually Impaired People: A Systematic Review. IEEE Access.

[6] Zhang H., Ye C. (2017). An indoor wayfinding system based on geometric features aided graph SLAM for the visually impaired. IEEE Trans. Neural Syst. Rehabil. Eng..

[7] Jafri R., Campos R.L., Ali S.A., Arabnia H.R. (2017). Visual and infrared sensor data-based obstacle detection for the visually impaired using the Google project tango tablet development kit and the unity engine. IEEE Access.

[8] Neto L.B., Grijalva F., Maike V.R.M.L., Martini L.C., Florencio D., Baranauskas M.C.C., Rocha A., Goldenstein S. (2016). A kinect-based wearable face recognition system to aid visually impaired users. IEEE Trans. Hum.-Mach. Syst..

[9] Ahmetovic D., Gleason C., Ruan C., Kitani K., Takagi H., Asakawa C. NavCog: A navigational cognitive assistant for the blind. Proceedings of the 18th International Conference on Human-Computer Interaction with Mobile Devices and Services.

[10] Apostolopoulos I., Fallah N., Folmer E., Bekris K.E. (2014). Integrated online localization and navigation for people with visual impairments using smart phones. ACM Trans. Interact. Intell. Syst. (TiiS).

[11] Hsieh Y.Z., Lin S.S., Xu F.X. (2020). Development of a wearable guide device based on convolutional neural network for blind or visually impaired persons. Multimed. Tools Appl..

[12] Barontini F., Catalano M.G., Pallottino L., Leporini B., Bianchi M. (2020). Integrating Wearable Haptics and Obstacle Avoidance for the Visually Impaired in Indoor Navigation: A User-Centered Approach. IEEE Trans. Haptics.

[13] Bai J., Liu Z., Lin Y., Li Y., Lian S., Liu D. (2019). Wearable travel aid for environment perception and navigation of visually impaired people. Electronics.

[14] Mancini A., Frontoni E., Zingaretti P. (2018). Mechatronic system to help visually impaired users during walking and running. IEEE Trans. Intell. Transp. Syst..

[15] Abita J.L., Stanford R., Carkhuff B. (1998). Alarm System for Blind and Visually Impaired Individuals. U.S. Patent.

[16] Searle C., Searle S., Holbrook J. (2016). Navigation Device for the Visually-Impaired. U.S. Patent.

[17] Saud S.N., Raya L., Abdullah M.I., Isa M.Z.A. (2021). Smart Navigation Aids for Blind and Vision Impairment People. Computational Intelligence in Information Systems.

[18] Messaoudi M.D., Menelas B.A.J., Mcheick H. (2020). Autonomous Smart White Cane Navigation System for Indoor Usage. Technologies.

[19] Tayyaba S., Ashraf M.W., Alquthami T., Ahmad Z., Manzoor S. (2020). Fuzzy-Based Approach Using IoT Devices for Smart Home to Assist Blind People for Navigation. Sensors.

[20] Chen Z., Liu X., Kojima M., Huang Q., Arai T. (2021). A Wearable Navigation Device for Visually Impaired People Based on the Real-Time Semantic Visual SLAM System. Sensors.

[21] Kuriakose B., Shrestha R., Sandnes F.E. (2020). Tools and Technologies for Blind and Visually Impaired Navigation Support: A Review. IETE Tech. Rev..

[22] Yuan D., Manduchi R. Dynamic environment exploration using a virtual white cane. Proceedings of the 2005 IEEE Computer Society Conference on Computer Vision and Pattern Recognition (CVPR’05).

[23] Tahat A. A wireless ranging system for the blind long-cane utilizing a smart-phone. Proceedings of the 2009 10th International Conference on Telecommunications.

[24] Bolgiano D., Meeks E. (1967). A laser cane for the blind. IEEE J. Quantum Electron..

[25] Milios E., Kapralos B., Kopinska A., Stergiopoulos S. (2003). Sonification of range information for 3-D space perception. IEEE Trans. Neural Syst. Rehabil. Eng..

[26] Chang W.J., Chen L.B., Chen M.C., Su J.P., Sie C.Y., Yang C.H. (2020). Design and Implementation of an Intelligent Assistive System for Visually Impaired People for Aerial Obstacle Avoidance and Fall Detection. IEEE Sens. J..

[27] Landa-Hernández A., Casarubias-Vargas H., Bayro-Corrochano E. (2013). Geometric fuzzy techniques for guidance of visually impaired people. Appl. Bionics Biomech..

[28] Li B., Muñoz J.P., Rong X., Chen Q., Xiao J., Tian Y., Arditi A., Yousuf M. (2018). Vision-based mobile indoor assistive navigation aid for blind people. IEEE Trans. Mob. Comput..

[29] Chen S., Yao D., Cao H., Shen C. (2019). A Novel Approach to Wearable Image Recognition Systems to Aid Visually Impaired People. Appl. Sci..

[30] Sainarayanan G., Nagarajan R., Yaacob S. (2007). Fuzzy image processing scheme for autonomous navigation of human blind. Appl. Soft Comput..

[31] Kanwal N., Bostanci E., Currie K., Clark A.F. (2015). A navigation system for the visually impaired: A fusion of vision and depth sensor. Appl. Bionics Biomech..

[32] Stoll C., Palluel-Germain R., Fristot V., Pellerin D., Alleysson D., Graff C. (2015). Navigating from a depth image converted into sound. Appl. Bionics Biomech..

[33] Pham H.H., Le T.L., Vuillerme N. (2016). Real-time obstacle detection system in indoor environment for the visually impaired using microsoft kinect sensor. J. Sens..

[34] Marston J.R., Loomis J.M., Klatzky R.L., Golledge R.G. (2007). Nonvisual route following with guidance from a simple haptic or auditory display. J. Vis. Impair. Blind..

[35] Guerrero L.A., Vasquez F., Ochoa S.F. (2012). An indoor navigation system for the visually impaired. Sensors.

[36] Heuten W., Henze N., Boll S., Pielot M. Tactile wayfinder: A non-visual support system for wayfinding. Proceedings of the 5th Nordic Conference on Human-Computer Interaction: Building Bridges.

[37] Velázquez R., Pissaloux E., Lay-Ekuakille A. (2015). Tactile-foot stimulation can assist the navigation of people with visual impairment. Appl. Bionics Biomech..

